# The Question of Proprietaries

**Published:** 1905-11

**Authors:** 


### The Question of Proprietaries.

The antagonism in certain quarters to proprietaries is rapidly reaching a phase of acute hysteria. Several excellent journals, sane and sound in every other particular, are making themselves ridiculous in their hysterical crusade against private formulas and proprietary remedies. They insist on the publication of a formula with each advertisement, but like those who "want to eat of the pudding, but save the pudding string," the formula can be any old thing, and some of the formulas printed can not fail to cause a smile. They are about as explicit as some old mammy's receipt for her famous hoe cake—"some o' dis, an' some o' dat, an' some o' dat an' some o' dis; den cook to suit you selbes, an' eat when eber you want ter." The formulas in a great many instances mean little or nothing, and their publication in the light of editorial contention is an insult to the readers of such journals. It is an all too evident attempt to appear righteous, and do—different.

The whole question of the warfare on so-called secret proprietaries smirks of sensationalism. In the multiplication of doctors and medical journals there is a constantly growing search for new subjects for agitation and new means for aggrandizement. There is always a class who, if they can not create, refuse to accept the order of things, and so devote their energies to breaking down what others have built. The same spirit makes the nihilist what he is, and robs many a man of the credit, honor or peace that should be his. It is responsible for most of the world's unhappiness, and while giving nothing to mankind, takes much away. The basis of it all is the human tendency toward ugly, jealous envy, bereft of the neutralizing desires to advance by work and worth.

Frankly, with all due respect to those who think differently, we have none of this soul-stirring antipathy to honest proprietary remedies. We maintain that modern therapeutics owe much to the pharmaceutical studies and researches which have produced so many of our leading proprietary preparations. Much of our present day knowledge concerning pharmacal products has come through commercial enterprises. Therefore we earnestly believe that a field of activity which has accomplished so much good, indirectly perhaps, but none the less surely, should not be condemned wholesale.

We make a distinction between the honest proprietary and the dishonest proprietary. The first is a preparation or combination of drugs which, after careful, painstaking study, has been found by its manufacturers to have a definite field of usefulness. It is submitted to the medical profession and its intelligent use enlarges or narrows its therapeutic value. A certain amount of information concerning its ingredients, if a combination of drugs, assuredly facilitates its intelligent us by the physician, but the specific quantity of each ingredient is not necessary, for it is from the combination as a whole that the chief result often comes. The manufacturer, who perhaps has spent hundreds or thousands of dollars in perfecting the formula and its special ingredients, can not be expected to give gratuitously the product of his time, thought and money. This may be proper in the next world, or some future Utopia, but the substitutor and unscrupulous pharmacist are too prevalent just at present to justify or even make advisable any such altruistic act. Then again it should be remembered that the manufacturer forces no one to use his preparation. They can take it, or leave it, just as they wish. Those who find it worthy should expect to pay a reasonable price, and if it is worthless it is dear at any price. Every manufacturer knows therefore that his product depends primarily on its real value. Advertising may create a temporary demand for anything, but true worth must also exist to establish a permanent one. This is the keynote of the whole matter—worth. If a preparation is worth anything and has a field of usefulness justified by experience, knowledge of its exact formula is not necessary to warrant its use. A physician who refuses to use a pharmaceutical product he does not know just how it was made, or just how much of everything it contains, is to be pitied for his mental strabismus. If he carries the principle to its logical conclusion, he will have to supervise or personally prepare everything given to his patients. The statements of his druggist or dispenser may be wrong and he is not doing his whole duty by his patients in not eliminating every uncertainty!

How absurd and far-fetched this all is. This old world is full of deceit and untruth, but we are not quite ready to let our haste lead us into the error of branding all men as liars. There is a lot of good and truth and honesty and trustworthiness among men after all. Consequently we are going on using such proprietaries as appeal to our judgment, needs and experience, and we claim the sole right of deciding the matter for ourselves. We are the last court of decision in regard to our own acceptance or rejection of any method, preparation or drug, and so long as no one suffers from the exercise of our own conscience and judgment, *no man can say us nay!*

The time is past when cult, school or pathy have any place in the intelligent practice of medicine. Every physician has a definite obligation in his work—to accomplish the most in the best possible way. No different ethics are needed for a practitioner of medicine than for any other honest man. Those who think differently can fight the matter out for themselves. For us there is only one code for all mankind—that of the humble Galilean. The Golden Rule, backed by an earnest attempt to make the most of what one is, to tell the truth, and to recognize no defeat but death, is code enough for any man.

In regard to dishonest proprietaries no argument or condemnation is necessary. They are not what they claim to be, they betray the trust they falsely create, and they are evils that should be fought. Experience is often necessary to discover them, but when discovered they should be given a wide berth.

In conclusion, we have only this short word to say: Honest proprietaries fill an important place in the modern treatment of disease. If they are honest and worthy, their measure is in results, and honest results count for the most in the practice of medicine today.

If not, what does?—*Vermont Medical Monthly.*

